# 

**Published:** 1853-07

**Authors:** 


### TO READERS AND CORRESPONDENTS.

**Communications intended for publication, and all letters relating to the business of the Journal, or containing remittances in money, should be directed to Drs. Harris and Blandy, Baltimore, Md.**

### *Medical and Dental Journals Received.*

1. *The Dental Register of the West.* Edited by James Taylor, M. D., D. D. S. Published by order of the Mississippi Valley Association of Dental Surgeons. April, 1853. *In Exchange.*

2. *The Dental News Letter.* April, 1853. *In Exchange.*

3. *Boston Medical and Surgical Journal.* Edited by J. V. C. Smith, M. D. April, May and June, 1853. *In Exchange.*

4. *The British and Foreign Medico-Chirurgical Review.* April, 1853. *In Exchange.*

5. *The American Journal of the Medical Sciences.* Edited by Isaac Hays, M. D., Surgeon to Wills Hospital, &c., April, 1853. *In Exchange.*

6. *The New York Dental Recorder.* Edited by C. C. Allen, M. D., Dentist, and A. Hill, D. D. S. April, May and June, 1853. *In Exchange.*

7. *The Ohio Medical and Surgical Journal.* Edited by Richard L. Howard, M. D. April, May and June, 1853. *In Exchange.*

8. *The Southern Medical and Surgical Journal.* Edited by J. P. Garvin, M. D. April, May and June, 1853. *In Exchange.*

9. *The New York Medical Gazette, and Journal of Health.* Edited by D. M. Reese, M. D., LLD. April, May and June, 1853. *In Exchange.*

10. *The New York Journal of Medicine and the Collateral Sciences.* Edited by S. S. Purple, M. D. March, April, May and June, 1853. *In Exchange.*

11. *The Medical Examiner.* Edited by F. G. Smith, M. D., and John Biddle, M. D. April, May and June, 1853. *In Exchange.*

12. *The Southern Journal of the Medical and Physical Sciences.* Edited by Drs. J. W. King, W. P. Jones, F. A. Ramsey, R. O. Currey and B. Wood. June and July, 1853. *In Exehange.*

13. *The New Jersey Medical Reporter, and Transactions of the New Jersey Medical Society.* Edited by Joseph Parish, M. D. April, May and June, 1853. *In Exchange.*

14. *The Western Lancet and Hospital Reporter.* Edited by L. M. Lawson, M. D., and George Mendenhall, M. D. Not received. *In Exchange.*

15. *The North Western Medical and Surgical Journal.* Edited by J. Evans, M. D., and E. G. Meek, A. M., M. D. April, May and June, 1853. *In Exchange.*

16. *The Western Journal of Medicine and Surgery.* Edited by L. P. Yandell, M. D., and T. S. Bell, M. D. April, May and June, 1853. *In Exchange.*

### *To Readers and Correspondents.*

17. *The New Hampshire Journal of Medicine.* Edited by EDWARD H. PARKER, A. M., M. D. April, May and June, 1853. *In Exchange.*

18. *The Charleston Medical Journal aud Review.* Edited by D. J. CAIN, M. D., and F. PEYREL PORCHER, M. D. December, 1852. January, February, March, April and May, 1853. *In Exchange.*

19. *The Buffalo Medical Journal* Edited by AUSTIN FLINT, M. D. April, May and June, 1853. *In Exchange.*

20. *The Provincial Medical aud Surgical Journal.* Edited by G. W. H. RANKIN, M. D., and J. H. WALSH, Esq. March, April, May and June, 1853. *In Exchange.*

21. *The London Lancet*, Journal of British and Foreign, Surgical and Chemical Sciences, Chemistry, Literature and News. Editor THOMAS WAKELEY, M. D., for London. Sub-Editors, J. H. BENNET, M. D., and T. WAKELEY, M. R. C. S. E. April, May and June, 1853. *In Exchange.*

22. *The Northern Lancet and Gazette of Legal Medicine.* A monthly Journal of Medicine and General Science, Criticism, and Medical Jurisprudence. Edited by HORACE NELSON, M. D., and FRANCIS J. D. AVIGNON, M. D. April, May and June, 1853. *In Exchange.*

23. *The Dublin Medical Press.* April, May and June, 1853. *In Exchange.*

24. *Virginia Medical and Surgical Journal.* Edited by Drs. GEORGE A. OTIS and H. L. THOMAS. May and June, 1853.

***Editorial Change.***—The Dental News Letter, heretofore conducted by Mr. McCurdy, of the firm of Jones, White & McCurdy, will hereafter, we understand, be under the editorial management of Professor J. D. White, M. D., already favorably known to the profession as the author of several able contributions to its literature. We welcome Dr. W. most cordially to the field of editorial labor, feeling assured that the News Letter, under his management, will not lose any of the popularity which it has hitherto so justly enjoyed.

***Omitted.***—The description which we designed giving in the present number of the Journal, of a method of mounting artificial teeth, introduced by Dr. Hullihen, is unavoidably omitted, as we were not able to get the cuts, necessary for its illustration, ready in time for this issue, which has unavoidably been delayed several weeks. The article will appear in the next number, which we hope to have out by the last of September or the first of October.

***The End of the Volume.***—Again, we have reached the close of the volume of the Dental Journal. We have zealously labored during the progress of it, to lay before the profession facts, both new and useful, and we believe we have succeeded in imparting much valuable information. We ask our readers to look back over what we have submitted to their consideration during the past year and to say whether we have or have not redeemed our promises made at the outset.

But there is another consideration of great importance to us, which is suggested by the close of the volume. The printer has claims upon us, the validity of which we must recognize. There is no avoiding the inexorable pay-day, and there is but one way in which we shall be enabled to meet it. Let our ***subscribers*** come up as punctually as our ***printer*** and we shall have an easy time of it. There is a large amount of back debts upon our list and we shall be most happy to transfer them to our cash-book.

### INDEX.

PAGE.

A.

Abscess of the tongue. By Mr. Coote, . . . . . 109
Advancement of dentistry, . 73
Allen, C. M., retro-pharyngeal abscess, . . . . 297, 432
Allen, John, review of Hunter's formulas, . . . . 280
Alumni of dental colleges, meeting of, . . . . . 500
Alveolar hemorrhage compress. By R. Reid, . . . 117
American Society of Dental Surgeons, . . . . . 159
Amercan Acad'y of Dental Science, 508
A moment's reflection, . . 511
Amputation of bony tumor of upper jaw, . . . . 457
Amputation of upper jaw for removal of tumor of the pharynx, 462
Anæsthetics, use of in ancient China, . . . . . 463
Ancient dental instruments, . 333
Antrum, disease of, produced by a fall, . . . . . 635
Arsenic, new form of, for destroying dental nerve, . . . 496
Artery, singular development of an, of the gums, . . . 664
Arthur, Robert, treatment of dental caries, . . . . . 3
Arthur, Robert, dental patents, 530
Artificial teeth and gums. By Wm. M. Hunter, . . . 44
Austen, P. H., valedictory, Baltimore College Dental Surgery, . 370
Avery, Mr., fissure of soft and hard palate, . . . . 129

B.

Baltimore College of Dental Surgery, annual commencement of, 505

PAGE.

Barker, T. H., cancrum oris and necrosis of inferior maxillary bone, 293
Bernard, M. C., absorption and respiration, . . . . 602
Bilisurio, John, inflammation of the pulps of ten teeth, . . . 430
Blood, effects of chloroform on the, 637
Bond, T. E., dental medicine, . 157
Bone, structure and development of. By John Tomes, . . 447
Bullock, W. G., excision of inferior maxillary bone for caries, . 640
Burpee, H. L., ophthalmitis from wearing a dental substitute, . 232
Byford, Wm. H., stomatitis materna, . . . . . 479

C.

Campbell, Henry, difficulties and privileges of the medical profession, . . . . . 155
Cancrum oris, . . 112, 293
Caries of the teeth, treatment of. By Robert Arthur, . . 3
Caries of the teeth, . . . 489
Caries, dental, treatment of. By W. H. Dwinelle, . . . 169
Carious teeth, effects of. By Abr. Robertson, . . . . 93
Carcinoma of eyelid, case of. By C. T. Meier, . . . . 290
Carpenter's Human Physiology, 324, 502
Carson, Joseph, valedictory, University of Pennsylvania, . 503
Carcinomatous tumor of uvula and fauces, . . . . . 124
Chemistry applied to dentistry. By A. S. Piggot, . . . . 248
Chemistry as a branch of medical education, . . . . 661
Chloroform, resuscitation from asphyxia, caused by, . . 474

PAGE.
Chloroform, effects of, on the blood, 637
Cleft palate, operation for. By John Gay, . . . 475
Cleveland, J. A., obituary of, . 335
Clinical phrase book, in English and German. By M. Johns, . 656
Coale, W. E., uterine displacements, . . . . . 657
Conscientious patients, . . 511
Convulsions cured by lancing gums. By V. M. Swazye, . 102
Crane, F. L., treatment of hemorrhage, . . . . . 428
Cushman, C. T., replacement of teeth, . . . 224, 597

D.

Davenport, J. W., mirror of dentistry, . . . . . 325
Dental education. By B. Wood, 28, 180, 331, 509
Dental medicine. By T. E. Bond, 157
Dental patents. By A. Hill, . 400
Dental patents. By G. W. Kendall, . . . . . 414
Dental patents. By Robt. Arthur, 530
Dental Surgeons, Mississippi Valley Association of, . . 77
Dental surgery, principles and practice of, . . . . . 157
Dental works in preparation for the press, . . . . 163
Dentine, anatomy and physiology of. By J. De Haven White, . 215
Dentist's Case Book. By J. L. Levison, . . . . 39
Dentition, first, mortality of children from. By J. L. Levison, . 360
Dentizione sapienza, ancora su la. By N. Martelli, . . . 659
Digestion and its relation to the teeth. By W. R. Handy, . 57
Diseases of children. By J. F. Meigs, . . . . 660
Druggist's receipt book, . . 325
Dwinelle, W. H., the sense of taste and effects of plates in the mouth, 103
Dwinelle, W. H., treatment of deep-seated caries, . . . . 169
Dwinelle, W. H., microscopes, . 236

E.

Editorial change, . . . . 664
Ellis, Geo. V., demonstration of anatomy, . . . . 502
End of the volume, . . . 664
Erosion of the enamel, . . . 498
Exchanges, a word to our, . . 327

PAGE.
Exostosis, dental, 162, 456, 600
Extraction of teeth. By J. Taylor, . . . . . 645

F.

Fall, disease of antrum produced by, . . . . . 635
Fauchard, . . 73, 162
Fees, professional. By E. Townsend, . . . . . 18
Fergusson, Mr., tic douloureux in inferior dental nerve, . . 443
Filing the teeth, . . . 495
Filling teeth, . . . . 490
Fowell's treatise on dentistry, . 658

G.

Gay, John, operation for cleft palate, . . . . . 475
Georgia, Medical Society of, transactions of third annual meeting, 155
Gold, gold-beating, &c. By R. N. Wright, . 243, 387, 551
Gold plate and solder. By B. Wood, 308
Gold, patent crystalline sponge, . 642
Gross, S. D., excision of superior maxillary bone, . 131, 262
Gums, singular development of an artery of, . . . 664

H.

Hancock, Mr., removal of superior maxilla, with large tumor, . 629
Handy, W. R., digestion, . 57
Headland, on the action of medicines in the system, . . 653
Hemorrhage from extraction of a tooth. By S. A. Saltonstall, . 90
Hemorrhage, treatment of. By F. L. Crane, . . . 428
Hemorrhage from lancing gums, death from, . . 461, 496
Hemorrhage from gums, . 496
Hemorrhage from extraction of teeth, . . . . . 496
Hemorrhage, extraordinary case of. By T. C. Harris, . . 599
Henle, J., rational pathology, . 657
Hill, R., dental patents, . . 400
Holden, L., manual of dissection of the human body, . . 505
Horn upon the head of a woman, 463
Hughes, J. G., introductory lecture, Iowa University, . . 503
Hullihen's method of treating teeth after exposure of lining membrane, . . . . . 160
Hullihen, S. P., method of mounting single teeth, . . 510

PAGE.
Hunter, Wm. M., new method of supplying artificial teeth and gums, . . . . . 44
Hunter's Formulas, review of. By John Allen, . . . 280

I.

Immobility of inferior maxilla, from abuse of mercury. By H. H. Toland, . . . . . 465
Irregularity of teeth. By E. G. Tucker, . . . . . 67

J.

Jaws and teeth in semi-barbarous races of men, . . . 121
Johns, M., clinical phrase book, in English and German, . . 656
Jones, Wm., reproduction of inferior maxillary bone . . . 592
Jordan, Henry, on the teeth, 154, 658
Jottings in a dentist's gold book. By E. Townsend, . . . 421

K.

Kendall, G. W., dental patents, . 414
Key, extraction of teeth with the, 491
Keyes, J. W., patents and patentees, . . . . . 259
Knox, R., manual of human anatomy, . . . . . 504
Kreonele, Edwin, exostosis on root of first lower molar, . 600

L.

Leigh, E., philosophy of medical science, . . . . . 649
Leigh, E., respiration subservient to nutrition, . . . 656
Letter from W. H. Dwinelle, . 512
Levison, J. L., dentist's case book, 39
Levison, J. L., salivary calculus in gouty persons, . . . 256
Levison, J. L., mortality of children from first dentition, . 360
Lining membrane, S. P. Hullihen's method of treating teeth after exposure of, . . . . 160
Live and learn, . . . . 167

M.

Martelli, N., ancora su la dentizione sapienza, . . . . 659
Maxilla, removal of half of lower, for secondary cancerous manifestations, . . . . 127
Maxillary bone, excision of superior. By S. D. Gross, . 131, 262
Maxilla, removal of left superior, with large tumor. By Mr. Hancock, . . . . . 629
Maxillary bone, excision of inferior, for caries. By W. G. Bullock, 640
McSherry on gun-shot wounds, . 155
Medical education, chemistry as a branch of, . . . . 661
Meier, C. T., carcinoma of eyelid, 290
Meigs, J. F. diseases of children, . 660
Microscopes. By W. H. Dwinelle, 236
Mississippi Valley Association of Dental Surgeons, eighth annual meeting, . . . . 77
Missouri, transactions of Medical Association of, . . . . 156
Mirror of dentistry. By J. W. Davenport, . . . 325
Morton's claim of the discovery of anæsthetic agents, . . 325
Movable seat dentist's chair, . 335
Muscular power, . . . . 165

N.

Necrosis of the maxillæ, . . 166
New Jersey Medical Institute, . 334
New Year, . . . . 334
New York Medical College announcement, . . . . 155
New York College of Dental Surgery, second annual announcement, . . . . 156
New York College Dental Surgery, commencement, . . 507

O.

Obituary, Dr. J. A. Cleveland, . 335
Odontalgia, its pathology and treatment. By J. P. Togg, . . 513
Old arts and sciences, . . 512
Omitted, . . . . . 664
Ophthalmitis from wearing a dental substitute. By H. L. Burpee, 232
Osseous union of teeth, . . 333
Ossification of dental pulp, . 510

P.

Palate, fissure of. By Mr. Avery, 129
Partridge, Mr., recurrence of osteo-cartilaginous tumor, . . 152
Patents and patentees. By J. W. Keyes, . . . . . 259
Patent crystalline sponge gold, . 642
Pathology, rational. By J. Henle, 657
Pennsylvania Medical Soc., transactions of, . . . . 156
Philadelphia College of Dental Surgery, commencement, . 507
Philosophy of medical science. By E. Leigh, . . . 649

PAGE.
Physician's visiting list, &c. . 156
Physiology, general and comparative. By Wm. B. Carpenter, . 502
Physiology of absorption and of respiration. By M. C. Bernard, . 602
Piggot, A. S., application of chemistry to dentistry, . . 248
Piggot, A. S., on saliva, . 337, 565
Pivot teeth, inserting. By A. T. Willard, . . . 497
Plate teeth on pivot. By S. A. Saltonstall, . . . 258
Poisonous chloroform, . . 498
Principles and practice of dental surgery, . . . 157
Prize essays, . . 168, 510
Professional merit. By S. D. Thompson, . . . 91
Purple of Cassius, . . 441

R.

Recipe for diseased gums, &c., . 500
Reid, R., alveolar abscess compress, . . . . 117
Removing teeth from plates, . 498
Replacement of teeth. By C. T. Cushman, . . 224, 597
Reproduction of inferior maxillary bone. By Wm. Jones, . 592
Respiration subservient to nutrition. By E. Leigh, . . 656
Retro-pharyngeal abscess. By C. M. Allen, . 297, 432
Reuben, L., introductory address, 503
Robertson, Abr., effects of carious teeth, . . . . 93

S.

Saliva, its constitution and function. By A. S. Piggot, . 337
Saliva, morbid changes of. By A. S. Piggot, . . . 565
Salivary calculus in gouty persons. By J. L. Levison, . . 256
Saltonstall, S. A., hemorrhage from extraction of a tooth, . 90
Saltonstall, S. A., plate teeth on pivot, . . . . . 258
Scirrhus of submaxillary gland, . 315
Singular death, . . . 164
Skull, wound of, . . . 314
Southern Journal of Medical and Physical Sciences, . . 504
Springing of plates, . . 490
St. Louis University, . . 156
Stomatitis materna. By Wm. H. Byford, . . . . 479
Styptic, new, . . . 454
Swazye, V. M., convulsions cured by lancing gums, . . 102

T.

Taylor, J., extraction of teeth, . 645
Teeth as objects of study, . . 492
Thackston, W. W. H., review of Drs. Gardette and Trenor, . 190
Thompson, S. D., professional merit, . . . . . 91
Tic douloureux in inferior dental nerve, . . . . 443
Tobacco, effects of, . . 163
Tobacco, a counterblast against, . 632
Togg, J. P., odontalgia, . . 513
Tomes, John, structure and development of bone, . . 447
Tongue, inflammation and abscess of, . . . . . 109
Townsend, E., professional fees, . 18
Townsend, E., jottings in the gold book of a dentist, . . 421
Transposition of teeth, . . 510
Tucker, E. G., irregularity of teeth, 67
Tumor, carcinomatous, of uvula and fauces, . . . 124
Tumor, recurrence of osteo-cartilagenous, connected with nasal process. By Mr. Partridge, . 152

U.

Uterine displacements. By W. E. Coale, . . . . 657

V.

Valedictory, Balt. College of Dental Surgery. By P. H. Austen, 370
Virginia Medical and Surgical Journal, . . . 335, 510

W.

Wells, Horace, discovery of the applicability of nitrous oxyd gas, &c., in surgical operations, . 323
Westcott, A., review of Trenor's remarks on professional education of dentists, . . . 154
White, J. De Haven, on dentine, 215
Willard, A. T., inserting pivot teeth, . . . . 497
Wood, B., collegiate dental education, . . 28, 180, 331
Wood, B., gold plate and solder, . 308
Wright, R. N., gold, gold-beating, &c., . . 243, 387, 551
Wythe's dose and symptom book, 324

Z.

Zahnheilkunde, von J. Liuderer, . 323

